# Food Intake and Nutrition in Children Undergoing Treatment for Pulmonary Tuberculosis: A Scoping Review

**DOI:** 10.3390/nu18162651

**Published:** 2026-08-13

**Authors:** Muhammad Rizal Martua Damanik, Guntari Prasetya, Bayu Satria Wiratama, Mohammad Hendra Setia Lesmana, Susaldi Susaldi

**Affiliations:** 1 Faculty of Medicine and Nutrition, IPB University, Bogor 16680, Indonesia; 2 Doctoral Study Program in Nutrition Science, Faculty of Medicine and Nutrition, IPB University, Bogor 16680, Indonesia; guntari.prasetya@stikesmitrakeluarga.ac.id; 3 Department of Nutrition, STIKes Mitra Keluarga, Bekasi 17113, Indonesia; 4 Department of Biostatistics, Epidemiology, and Population Health, Faculty of Medicine, Public Health, and Nursing, Universitas Gadjah Mada, Yogyakarta 55281, Indonesia; bayu.satria@ugm.ac.id; 5 Department of Mental and Community Nursing, Faculty of Medicine, Public Health and Nursing, Universitas Gadjah Mada, Yogyakarta 55281, Indonesia; hendralesmana090294@ugm.ac.id; 6 Faculty of Medicine, Public Health, and Nursing, Universitas Gadjah Mada, Yogyakarta 55281, Indonesia; susaldi@mail.ugm.ac.id; 7 Faculty of Health Sciences, Universitas Indonesia Maju, Jakarta 12610, Indonesia

**Keywords:** children, food intake, nutrition, pulmonary tuberculosis, scoping review

## Abstract

Background/Objectives: Children account for ∼11% of global TB cases. Adequate nutrition improves risk prediction, treatment outcomes, and helps identify children for TB-preventive treatment. We aimed to identify current evidence on food intake and nutrition, compare it with guidelines, and describe how nutrition is integrated into care for children with pulmonary TB. Methods: We a scoping review adhering to Preferred Reporting Items for Systematic reviews and Meta-Analyses extension for Scoping Reviews (PRISMA-ScR) of peer-reviewed and gray literature from January 2013 to March 2026. Results: We found limited evidence: 16 research articles, six supplementary records, and two guideline documents. Evidence on nutrition in pediatric pulmonary TB remains insufficient. Many studies included nutritional status in clinical assessment. Few studies examined household food assistance. Conclusions: Rigorous studies are needed to build evidence and translate it into practice to improve outcomes and prevent malnutrition in children with pulmonary TB.

## 1. Introduction

Tuberculosis (TB) remains the leading cause of illness and death from a single bacterial infection, *Mycobacterium tuberculosis*. In 2024, an estimated 10.7 million people [95% UI: 9.9–11.5 million] developed TB and 1.23 million died [95% UI: 1.13–1.33 million]. Of those, 54% were men, 35% women, and 11% children. About 2 million children and adolescents aged 0–19 years develop TB annually [1,2]. Clinical outcomes like post-TB lung dysfunction and neurological disability in children and adolescents are often under-recognized and poorly managed in low-resource settings [3,4].

World Health Organization’s 2013 guideline on nutritional care for TB patients recommends nutritional assessment, counseling, management of acute malnutrition, micronutrient supplementation, and support for household contacts [5]. In 2025, WHO convened a guideline development group to update recommendations on TB and undernutrition based on new evidence [6].

Indonesia has the world’s second-highest TB burden [2]. A study in Kuningan, West Java, found that TB elimination modeling in Indonesia must go beyond biomedical interventions to include behavioral, environmental, and psychosocial factors, including nutrition care [7]. This review identifies current evidence on food intake and nutrition, compares it with existing guidelines, and describes how these factors are integrated into care for children with pulmonary TB.

## 2. Materials and Methods

We conducted a scoping review of peer-reviewed and gray literature from Google. Using Arksey and O’Malley (2005) framework allowed us to explore food intake and nutrition in children undergoing treatment for pulmonary tuberculosis, including research databases (PubMed and Scopus) [8]. Reporting followed Preferred Reporting Items for Systematic reviews and Meta-Analyses extension for Scoping Reviews (PRISMA-ScR) guidelines [9] (see File S1).

### 2.1. Inclusion and Exclusion Criteria

We aimed to identify recent evidence on food intake and nutrition for children undergoing pulmonary TB treatment. We included observational and experimental studies on interventions to improve children’s nutritional status and the link between nutrition, disease, and treatment. Studies had to address “pediatric pulmonary TB treatment” and “nutrition for TB in children.” Both dietary and supplementation interventions were included, but studies on children with acute-phase pulmonary TB were excluded.

Searches covered January 2013 to March 2026, English-language only. This period starts 13 years after WHO’s 2013 guidance on TB nutrition care. We prioritized findings from this period in the discussion.

### 2.2. Search and Screening Process

We searched Scopus and PubMed using keywords combining “pulmonary tuberculosis” or “tuberculosis”, and terms like “food,” “nutrition,” “dietary intake,” and “food intake” (see File S2).

We exported titles and abstracts, removed duplicates, and had one reviewer exclude irrelevant records. Two reviewers independently screened the remainder, resolving discrepancies with co-authors. Full texts were then reviewed by two reviewers; unresolved disagreements went to a third reviewer.

### 2.3. Gray Literature Search

We searched Google for gray literature on nutritional TB care, applying the same criteria and limiting results to the first 10 pages. We also manually checked best-practice documents identified in databases and Google.

### 2.4. Analysis

Data were synthesized narratively. Studies on nutritional status, food intake, and nutrition interventions for children with pulmonary TB were grouped by focus area. This identified patterns, strengths, and limitations in nutritional management. Findings were shared with co-authors and experts to refine interpretations.

## 3. Results

**Figure 1:**
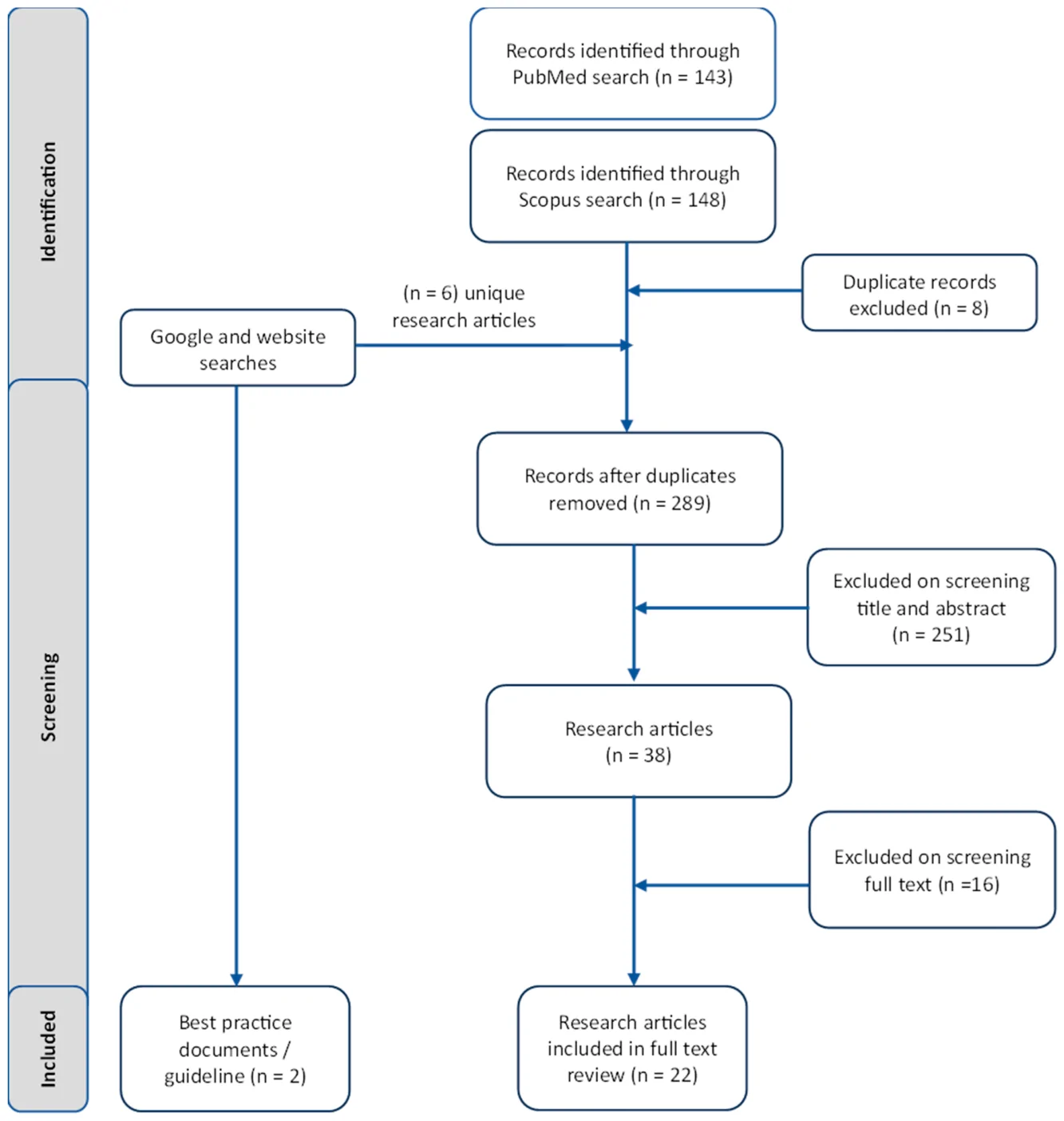
Process and outcome of identification and screening of research results.

We reviewed 16 full-text articles from database searches [10,11,12,13,14,15,16,17,18,19,20,21,22,23,24,25], six studies from gray literature [26,27,28,29,30,31], and two guideline documents [5,6] (Figure 1). They included 14 observational quantitative studies—6 cross-sectional, 3 prospective cohorts, 3 retrospective cohorts, and 2 case reports/case-controls, 2 qualitative observational studies, and 6 experimental studies—5 randomized controlled trials and 1 quasi-experimental study. Full text articles included in the review are also presented in the File S3. Based on WHO’s Nutritional Care and Support for Patients with Tuberculosis [5] and Consolidated TB Guidelines [6], we identified and described evidence on how nutrition affects children undergoing pulmonary TB treatment. Results are organized by guideline recommendations and the supporting studies.

### 3.1. Nutritional Status and Pediatric Tuberculosis

Nutritional status is critical in pediatric TB. Studies show more children with TB are underweight than symptomatic or healthy controls [11]. Machrumnizar et al. [10] found 64.7% of children aged 0–14 years with TB were malnourished. In adults, malnutrition and TB are mutually reinforcing: malnutrition raises the risk of active TB 6–10 times, and TB worsens malnutrition [32,33]. Addressing nutrition and dietary intake in children with pulmonary TB requires considering factors that affect nutritional status and recovery.

Malnutrition and micronutrient deficiencies are key challenges. Children with pulmonary TB are prone to malnutrition. High rates of undernutrition have been reported among pediatric TB patients [12]. Deficiencies in protein, vitamins A, D, E, and minerals like calcium, zinc, and selenium further impair immunity and recovery. We reviewed studies on nutritional status in children with pulmonary TB (Table 1).

Table 1 synthesizes studies on the link between nutritional status and pulmonary TB in children across countries, designs, and populations. An Indonesian cross-sectional study found 79% of children 0–14 years with pulmonary TB were malnourished [10]. Large Indian studies report TB in severely malnourished children, indicating malnutrition is a major TB risk factor [12,13]. A community study in Indian tribal populations found underweight, stunting, and wasting increased with age in children with pulmonary or extrapulmonary TB.

Cohort and longitudinal studies show nutritional status improves slowly in children with pulmonary TB. Based on the WHO child growth standards, malnutrition remained prevalent among children with tuberculosis even after completion of anti-tuberculosis treatment. From a population-level perspective, assessment of the entire cohort using WHO growth indicators revealed that BMI-for-age values remained below the normal reference cut-offs, indicating persistent nutritional deficits despite treatment [14]. The CHAIN Network cohort found children with clinically diagnosed TB had poorer anthropometric outcomes than non-TB children at enrolment and post-discharge [18].

Other studies highlight the bidirectional TB–nutrition relationship. Nutritional status often improves after anti-TB treatment, but outcomes vary by TB type and comorbidities [15,16,26,27]. Nutritional status of children aged 0–5 years diagnosed with TB declines due to decreased appetite, leading to weight loss; hence, a balanced comparison was found between children with good and poor nutritional status [31]. Caregiver factors also matter: improving mothers’ caregiving capacity can improve children’s nutrition [29]. De Geus et al. [11] found that children with TB had lower height-for-age z-scores than symptomatic and healthy controls, confirming chronic undernutrition.

In summary: (1) Undernutrition and wasting are highly prevalent in children with pulmonary TB. (2) TB worsens nutrition through poor appetite and higher metabolic demands. (3) Nutrition can improve with TB treatment but recovery is often incomplete and influenced by other factors. (4) Dietary, socioeconomic, and caregiver factors affect outcomes.

### 3.2. Food and Nutrient Intake in Children with Pulmonary Tuberculosis

Studies on food intake in children with pulmonary TB remain scarce. We listed studies on dietary intake, nutrition interventions, and supplementation provided to children during pulmonary TB treatment in Table 2.

**Figure 2:**
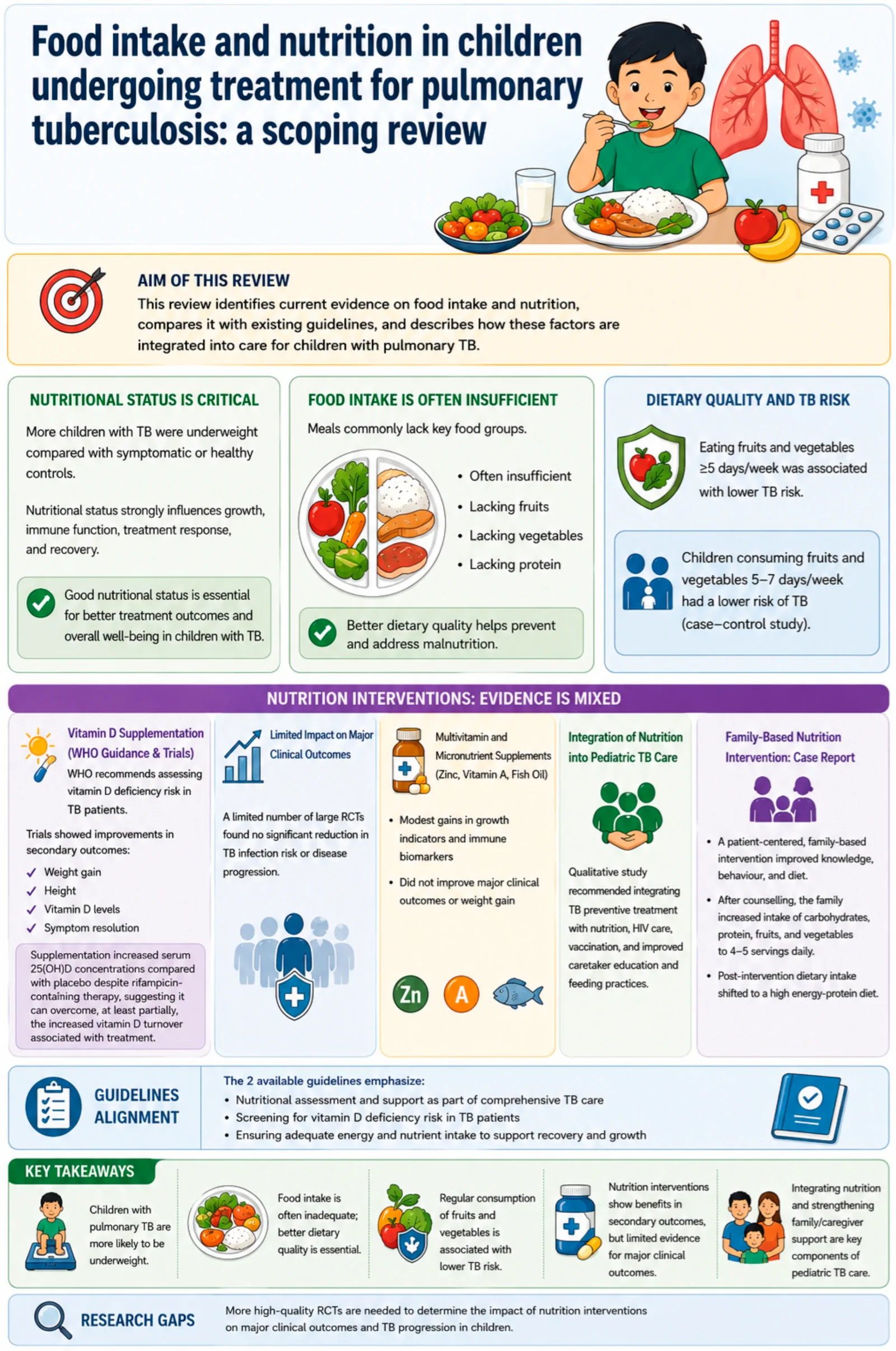
Key findings: Nutrition in children undergoing TB treatment.

Available studies examined dietary intake, nutritional status, and nutrition interventions in children with pulmonary TB (Figure 2). Food intake was often insufficient, with meals lacking fruits, vegetables, and protein [14]. Better dietary quality helps prevent and address malnutrition. A cross-sectional study found eating fruits and vegetables ≥5 days/week lowered TB risk, highlighting the value of a balanced diet [17].

Evidence on nutrition interventions is mixed (Figure 2). Vitamin D supplementation is the most studied [22,25,28]. WHO guidelines recommend checking vitamin D deficiency risk in TB patients [5,6]. Trials showed improvements in secondary outcomes like weight gain, height, vitamin D levels, and symptom resolution. The study demonstrated that supplementation successfully increased serum 25(OH)D concentrations compared with placebo despite ongoing rifampicin-containing therapy, suggesting that pharmacological vitamin D supplementation can overcome, at least partially, the increased vitamin D turnover associated with treatment [22].

However, large RCTs found no significant reduction in TB infection risk or disease progression [23,25]. Multivitamin and micronutrient supplements—zinc, vitamin A, fish oil—offered modest gains in growth indicators and immune biomarkers but did not improve major clinical outcomes or weight gain [19].

A qualitative study recommended integrating TB preventive treatment with nutrition, HIV care, vaccination, and improved caretaker education and feeding practices [24]. An Indonesian case report showed that a patient-centered, family-based intervention improved knowledge, behavior, and diet. After counseling, the family increased intake of carbohydrates, protein, fruits, and vegetables to 4–5 servings daily. Post-intervention dietary intake shifted to a high energy-protein diet [30].

## 4. Discussion

This scoping review summarizes current evidence on food intake and nutrition in children treated for pulmonary TB. Pulmonary TB involves the lung parenchyma or tracheobronchial tree. For surveillance, cases with both pulmonary and extrapulmonary TB are counted as pulmonary TB [34]. The review also reveals geographical and contextual differences in how countries integrate nutrition into the End TB Strategy.

### 4.1. General Nutritional Status

The mapped evidence on the nutritional status of children with pulmonary tuberculosis was derived predominantly from observational studies, including cross-sectional, cohort, retrospective, and longitudinal designs, whereas no randomized controlled trials (RCTs) specifically evaluated overall nutritional status as a primary outcome. Consequently, the evidence consistently demonstrates an association between pulmonary TB and poor nutritional status but provides limited ability to infer causality. Across diverse settings, observational studies consistently reported a high prevalence of undernutrition, persistent deficits in anthropometric indicators (particularly BMI-for-age and height-for-age), and an association between poor nutritional status and adverse treatment outcomes. Cohort and longitudinal studies further suggested that although weight may improve during anti-tuberculosis treatment, deficits in linear growth and overall nutritional recovery frequently persist. Collectively, these findings strengthen the consistency of the association across populations, but the predominance of observational designs means that the overall certainty of evidence remains moderate rather than high.

Many reported nutritional assessments during treatment align with WHO’s 2025 guideline recommendation for “nutritional assessment and counseling in people with TB and their household contacts” [6]. Study from Ethiopia reported by Burusie et al. [35] further suggests that persistent malnutrition in children younger than 10 years with tuberculosis is associated with an increased risk of mortality. Because stunting reflects the cumulative effects of long-term undernutrition, BMI-for-age and height-for-age may not be the most sensitive indicators for monitoring nutritional recovery in children with tuberculosis, particularly among those with pre-existing chronic malnutrition.

### 4.2. Specific Nutrients and Nutritional Interventions

Evidence regarding nutritional interventions incorporated several RCTs, providing stronger evidence than the observational studies for intervention effectiveness. The RCTs primarily evaluated vitamin D supplementation, micronutrient combinations (zinc, vitamin A, and fish oil), or multivitamin supplementation. However, their findings were heterogenous. Some randomized trials demonstrated improvements in selected clinical outcomes, symptom resolution, or nutritional status among vitamin D-deficient children [22,28], whereas other well-designed placebo-controlled trials found no significant benefit on TB incidence, weight gain, or treatment outcomes [23,25]. Observational and qualitative studies complemented these findings by identifying inadequate dietary intake, poor dietary diversity and barriers to nutrition service delivery, but these studies primarily generated contextual evidence rather than establishing intervention efficacy [14,15,16,17,30]. Therefore, while RCTs contribute higher-level evidence for specific nutritional interventions, the current evidence base remains insufficient to support universal micronutrient supplementation for all children with pulmonary TB. Instead, the available evidence favors individualized nutritional assessment and targeted interventions based on documented nutritional deficiencies and dietary needs.

### 4.3. Implications for Clinical Practice

The findings suggest that nutritional management should be integrated into routine pediatric TB care rather than being limited to general dietary counseling. Regular nutritional assessment using standardized anthropometric indicators (weight-for-age, height-for-age, BMI-for-age, and MUAC where appropriate) should be conducted at TB diagnosis, during treatment, and after treatment completion to identify children with persistent nutritional deficits [11,14,15,18]. Given the recurrent observation of inadequate dietary intake across the included studies, individualized dietary counseling should be complemented with practical interventions such as provision of nutrient-dense foods, high-energy/high-protein meal planning, and linkage to food assistance programs for food-insecure households [14,17,24,30].

Evidence from the limited number of randomized controlled trials suggests that routine micronutrient supplementation cannot yet be universally recommended for all children with pulmonary TB. Instead, micronutrient interventions, particularly vitamin D supplementation, should be considered in children with documented deficiency or those at high risk of deficiency, consistent with current clinical guidelines [6]. Further intervention strategies should also evaluate food-based approaches and multiple-micronutrient interventions in adequately powered randomized trials.

Micronutrient supplementation is widely used for children with TB. Studies show vitamin D, vitamin A, zinc, and fish oil benefit pediatric pulmonary TB patients regardless of nutritional status by improving nutrition and immune response [19]. WHO (2025) guideline states: Nutritional interventions should be given to all TB patients with undernutrition as part of TB care. In food insecurity, food baskets with multiple micronutrients should be given to all TB households [6].

This review emphasizes the need for standard nutrition guidelines and protocols for children with pulmonary TB, including nutritional assessment and interventions like food assistance and micronutrient supplementation. More research is needed on nutrition program effectiveness for preventing malnutrition and supporting TB treatment. Nutrition frameworks must move from research into clinical practice. Health leaders and practitioners should use dynamic, evidence-based, integrated, patient-centered, and sustainable models for children undergoing TB treatment.

### 4.4. Implications for Public Health Policy

The findings support integrating nutritional services into national pediatric TB programs. Public health authorities should incorporate routine nutritional screening, growth monitoring, and dietary assessment into childhood TB management protocols, particularly in high-burden settings where undernutrition and TB frequently coexist.

Multisectoral collaboration between TB control programs, maternal and child health services, nutrition programs, and social protection agencies is essential to address the underlying determinants of malnutrition. WHO defines food assistance as interventions that improve household food access, including direct food provision, cash transfers, food vouchers, and social protection programs. Few sources described intervention studies on food assistance for households with pediatric TB [6].

Qualitative studies also highlight family/home-based approaches as a strategy for TB preventive treatment [24,31]. Further research is needed to build evidence on implementing and evaluating nutrition in integrated care for children with pulmonary TB. Researchers and health systems should identify local barriers and facilitators and develop tailored strategies to support TB preventive treatment.

Strengthening the capacity of primary healthcare workers through standardized training on pediatric TB nutrition, nutritional risk assessment, and counseling may improve implementation of integrated care. Community health workers can also play an important role in monitoring dietary adherence, supporting caregivers, and facilitating referrals for nutritional rehabilitation.

### 4.5. Strengths and Limitations

Strengths: This scoping review followed PRISMA-ScR guidelines with a clear protocol, comprehensive search of peer-reviewed and gray literature, independent screening and extraction, and thorough documentation. Guidelines were also compared to current evidence.

Limitations: Limited to English publications, few experimental studies leading to low evidence level, and restricted to public sources which may miss unpublished implementation insights.

### 4.6. Research Priorities

The current evidence highlights several important research gaps.

- More multicenter randomized controlled trials are needed to evaluate the effectiveness of specific nutritional interventions—including food supplementation, balanced energy–protein supplementation, lipid-based nutrient supplements, multiple micronutrient formulations, and vitamin D—in improving both nutritional recovery and TB treatment outcomes.
- Future studies should standardize anthropometric outcomes and nutritional biomarkers to facilitate comparison across studies.
- Longer follow-up beyond treatment completion is needed to determine whether nutritional deficits, particularly stunting and impaired linear growth, persist after microbiological cure.
- Cost-effectiveness studies should evaluate the feasibility of integrating nutritional interventions into routine childhood TB services, particularly in low- and middle-income countries.
- Implementation research should identify effective delivery models for integrating nutrition services with TB care at the primary healthcare and community levels.

## 5. Conclusions

Research on food intake and nutrition in children with pulmonary TB is still developing. WHO has released practice guidelines, but evidence remains inconclusive on how nutrition supports children’s survival against *Mycobacterium tuberculosis*. Local context must be considered as barriers and opportunities by involving health system decision-makers, patients, and families. Further research is needed to build evidence on inputs, nutrition care processes, optimal nutrition and immunity outputs, and sustainable nutrition care outcomes.

## Figures and Tables

**Table 1:** Synthesis of literature on nutritional status in children with pulmonary TB.

Author	Year	Country	Study Design	Subject	Outcomes
Ghosh et al. [14]	2026	India	Longitudinal field-based study	Children 0–18 years (50% had extrapulmonary TB).	Persistent malnutrition (≤1Z score) for BMI for age (*n* = 17 at baseline to 14 at endline) and ≤2Z for height for age (*n* = 20 at baseline to 16 at endline), while weight-for-age (*n* = 25 at baseline to 24 at endline) improved from −0.04 Z to −0.02Z.
Machrumnizar et al. [10]	2025	Indonesia	Cross-sectional study	156 children 0–14 years	113 of 156 (72.4%) were diagnosed with pulmonary TB, 90 of 113 (79%) were malnutrition.
de Geus et al. [11]	2025	South Africa	Prospective cohort study	372 children 0–13 years	153 (41%) children with TB, more children with TB were underweight than symptomatic and healthy controls. Median HAZ of children with TB was lower compared to symptomatic and healthy controls (*p* = 0.0037).
Chisti et al. [18]	2025	Bangladesh and Uganda	The CHAIN Network cohort study	A total of 365 young children (2–23 months old)	Children with clinically diagnosed TB were more likely to be malnourished at enrolment, and anthropometric Z-scores were significantly lower among children classified as clinically diagnosed compared to unlikely TB throughout the post-discharge period.
Dewi et al. [26]	2025	Indonesia	Comparative analytical design with a cross-sectional approach	A total of 42 children 1–5 years.	Differences between the nutritional status of toddlers suffering from pulmonary tuberculosis before and after OAT treatment.
Saraswati et al. [31]	2025	Indonesia	Qualitative case-study design	5 primary informants, (mothers of children aged 0–5 years diagnosed with TB), and 3 supporting informants.	Nutritional status declines due to decreased appetite, leading to weight loss; a balanced comparison was found between children with good and poor nutritional status.
Amelia & Kaswandani [27]	2024	Indonesia	Retrospective, cross-sectional study	A total of 207 patients (0–18 years)	Notable improvements in nutritional status after two months of treatment, with a decrease in malnutrition and an increase in good nutritional status; the type of TB and the presence of comorbidities influence the outcomes of nutritional status during anti-TB treatment.
Umijati & Andajani [29]	2024	Indonesia	Cross-sectional study	A total of 39 mothers whose family member had TB	TB in children was influenced by their nutritional status. Children’s nutritional status can be improved by improving mothers’ ability to care for sick children.
Singh et al. [12]	2024	India	Multicenter cross-sectional study	A total of 4356 severely malnourished children	189 (4.3%) had pulmonary TB.
Singh et al. [13]	2021	India	Multicenter study: retrospective chart review	A total of 2121 children with SAM across 41 nutrition rehabilitation centers.	10 of 56 children with SAM who have been screened were confirmed to have pulmonary TB.
Tezol et al. [15]	2019	Turkey	Retrospective cohort study	A total of 77 children (0–14 years) diagnosed with TB	Nutrition status monitoring, correcting nutritional deficiencies and failures can be neglected in course of anti-tuberculosis treatment
Gurung et al. [16]	2018	Nepal	Cross-sectional descriptive study	A total of 133 TB patients taking anti-tubercular drug (*n* = 18, 13.5% in adolescent age, 10–20 years; *n* = 115, 86.5%, ≥20 years).	Food frequency, TB types, calorie intake, and nutritional status at the time of registration were significantly associated with the recent nutritional status of the participants.
Chiang et al. [20]	2016	Peru	Retrospective cohort study	A total of 232 TB patients (≤15 years)	Independent predictors of death/treatment failure were severe disease and WAZ ≤ −1.

Notes: Number of studies included: 13. CHAIN, The Childhood Acute Illness and Nutrition; TB, tuberculosis; OAT, oral anti-tuberculosis; HAZ, height-for-age z-scores; WAZ, weight-for-age z-scores; SAM, severe-acute-malnutrition.

**Table 2:** Synthesis of literature on food intake and nutrition intervention or supplementation in children with pulmonary TB.

Author	Year	Country	Study Design	Subject	Outcomes
Ghosh et al. [14]	2026	India	Longitudinal field-based study	Children 0–18 years (50% had extrapulmonary TB).	The food diary indicated that children (*n* = 21) missed an average of one meal per day, particularly protein and green vegetables.
Khairiyadi et al. [28]	2025	Indonesia	Prospective cohort design, a double-masked, individually randomized, placebo-controlled trial.	52 patients (aged 1–14 years), divided into 26 patients in the treatment group (OAT + vitamin D) and 26 patients in the control group (OAT)	The study’s findings in the control group revealed significant differences in the average increase in body weight (2.14 kg), height (3.48 cm), hemoglobin levels (0.93 mg/dL), lymphocyte count (5.57%), and vitamin D levels (6.29 ng/mL). The treatment group (1.000 IU daily supplementation of cholecalciferol) exhibited a mean increase in body weight of 2.48 kg, height of 4.39 cm, a hemoglobin level of 0.15 mg/dL, and vitamin D level of 5.97 ng/mL. From a clinical perspective, administering vitamin D can increase body weight and weight discrepancy and improve the nutritional status of children with tuberculosis. Statistically, administering vitamin D does not impact the nutritional condition of children with TB.
Salazar-Austin et al. [24]	2024	Ethiopia	Formative qualitative research, a cross-sectional design	A total of 60 stakeholders 15 program managers, 15 TB providers, and 15 HEWs, 15 caregivers of a child <15 years old who was exposed to TB in the last two years who was evaluated and offered either TPT or TB treatment	TB services be integrated into other home-based services, including HIV, nutrition, and vaccination services to reduce workload on the already overstretched health extension workers.
Tamara et al. [22]	2022	Indonesia	Randomized double-blind controlled trial	A total of 84 patients, aged 6 to 18 years old, newly diagnosed with pulmonary TB and vitamin D insufficiency	Vitamin D is beneficial in improving fever and cough resolution, and improving nutritional status in children with pulmonary TB and vitamin D insufficiency.
Oktofani & Zuraida [30]	2021	Indonesia	Case report	Pediatric patient (11 years old) with a newly diagnosed of pulmonary tuberculosis	There are changes in behavior and knowledge of healthy life and dietary habit in patients and families of patients after an intervention based on evidence-based medicine that is patient centered, family approach: Patient’s diet does not implement a balanced nutritional diet, Patient does not like vegetables and fruits, only likes certain types of fruit. Patient and family was given counseling regarding balanced nutritional diets and high-calorie, high-protein diets for patients
Ganmaa et al. [25]	2020	Mongolia	Randomized controlled trial	Children who had negative results for M. tuberculosis infection according to the QuantiFERON-TB Gold In-Tube assay (QFT) to receive a weekly oral dose of either 14,000 IU of vitamin D3 (*n* = 4418) or placebo (*n* = 4433) for 3 years.	Vitamin D supplementation did not result in a lower risk of TB infection, TB disease, or acute respiratory infection than placebo among vitamin D–deficient schoolchildren in Mongolia
Olofin et al. [23]	2016	Tanzania	Randomized controlled trial	Active tuberculosis in HIV-exposed children up to 2 years of age (*n* = 2358).	Multivitamin supplementation did not significantly alter the risk of active TB.
Franke et al. [17]	2014	Peru	Case–control study	194 case–control pairs: 16% of cases were <2 years of age, 32% were 2–5 years of age; 21% were 6–10 years of age and 31% were >10 years of age.	Consumption of fruits or vegetables ≥5 days per week was associated with nearly a 60% lower disease rate after adjusting for meat and fish intake.
Lodha et al. [21]	2014	India	Randomized, double-blind, placebo-controlled trial.	A total of 403 children *n* = 179 children or 44.4% with newly diagnosed intrathoracic TB.	Micronutrient supplementation did not modify the weight gain or clearance of lesions on CXR, but may improve height gain in children with intrathoracic TB in children.
Nenni et al. [19]	2013	Indonesia	Quasi- experimental study	A total of 22 children (aged 5–14 years) with a positive acid-fast bacilli (AFB) smear. The subjects were randomly divided into two groups; group I was given the supplement for one month and ATD treatment following WHO guidelines, while group II was only given ATD treatment.	Both groups had a significant increase in BMI (*p* ≤ 0.001) post-treatment compared to pre-treatment. Supplementation with zinc, vitamin A and fish oil is associated with a significant increase in leptin levels and a significant decrease in TNF-α levels among children treated for TB.

Notes: Number of studies included: 10; ATD, anti-tuberculosis drug; BMI, body mass index; CXR, chest-x-rays; HEWs, health extension workers; HIV, human immunodeficiency virus; TB, tuberculosis; TPT, TB preventive treatment.

## Data Availability

The original contributions presented in this study are included in the article/Supplementary Material. Further inquiries can be directed to the corresponding author.

## Supplementary Materials

The following supporting information can be downloaded at: https://www.mdpi.com/article/10.3390/nu18162651/s1, File S1: Preferred Reporting Items for Systematic reviews and Meta-Analyses extension for Scoping Reviews (PRISMA-ScR) Checklist; File S2: Full electronic search strategy for the PubMed and Scopus database; File S3: Full text research articles included in the review, grouped by the outcomes of nutritional status, food intake, micronutrient supplementation, and nutrition in children with pulmonary TB.

## Abbreviations

The following abbreviations are used in this manuscript:

TB	Tuberculosis
WHO	World Health Organization
PRISMA-ScR	Preferred Reporting Items for Systematic Reviews and Meta Analysis Extension for Scoping Reviews
