# Supplementary material for: Food Intake and Nutrition in Children Undergoing Treatment for Pulmonary Tuberculosis: A Scoping Review
Source: Nutrients. 2026 Aug 13;18(16):2651. doi: 10.3390/nu18162651 (PMC13516705; doi:10.3390/nu18162651)
Supplement: Supplementary file 1 [file nutrients-18-02651-s001.zip › File S2.pdf]

File S2: full electronic search strategy for the PubMed and Scopus database

We searched on the PubMed database using multiple keyword combinations related to food and nutrition intake and pulmonary tuberculosis in children as outlined in Table 1. We limited the search to between January 2013 and March 2026 (with the most recent search conducted on 10 March 2026) and to English-language manuscripts.

Table 1 – Full search strategy on PubMed database

PubMed search terms*	Number of records
nutrition AND “tuberculosis”	91
food OR nutrition AND “tuberculosis”	143
nutrition AND “pulmonary-tuberculosis”	21
food OR nutrition AND “pulmonary-tuberculosis”	15
food-intake AND “tuberculosis”	9
dietary-intake AND “tuberculosis”	6
food-intake AND “pulmonary-tuberculosis”	2

*Limits and filters applied:

- Date range: 01 January 2013 – 10 March 2026
- Articles: Case Reports, Clinical Study, Clinical Trial, Comparative Study, Observational Study, Randomized Controlled Trial
- Language: English
- Humans
- Child: birth-18 years
- Exclude preprints

Table 2 – Full search strategy on Scopus database

Scopus search terms*	Number of records
food OR nutrition AND “tuberculosis”	148

*Limits and filters applied:

- Date range: 01 January 2013 – 10 March 2026
- Articles: Case Reports, Clinical Study, Clinical Trial, Comparative Study, Observational Study, Randomized Controlled Trial
- Language: English
- Humans
- Child: birth-18 years
