# Supplementary material for: Food Intake and Nutrition in Children Undergoing Treatment for Pulmonary Tuberculosis: A Scoping Review
Source: Nutrients. 2026 Aug 13;18(16):2651. doi: 10.3390/nu18162651 (PMC13516705; doi:10.3390/nu18162651)
Supplement: Supplementary file 1 [file nutrients-18-02651-s001.zip › File S3.pdf]

File S3. Full text research articles included in the review, grouped by the outcomes of nutritional status, food intake, micronutrient supplementation, and nutrition in children with pulmonary TB

	First author, year	Setting/ Country	Design	Outcomes
Nutritional status	Ghosh et al. 2026	India	Longitudinal field-based study	Persistent malnutrition (<-1Z score) for BMI for age (n=17 at baseline to 14 at endline) and <-2Z for height for age (n=20 at baseline to 16 at endline), while weight-for-age (n=25 at baseline to 24 at endline) improved from -0.04 Z to -0.02 Z.
	Machrumnizar et al. 2025	Indonesia	Cross-sectional study	113 of 156 (72.4%) were diagnosed with pulmonary TB, 90 of 113 (79%) were malnutrition.
	de Geus et al. 2025	South Africa	Prospective cohort study	153 (41%) children with TB, more children with TB were underweight than symptomatic and healthy controls. Median HAZ of children with TB was lower compared to symptomatic and healthy controls (*P* = 0.0037).
	Chisti et al. 2025	Bangladesh & Uganda	The CHAIN Network cohort study	Children with clinically diagnosed TB were more likely to be malnourished at enrolment, and anthropometric Z-scores were significantly lower among children classified as clinically diagnosed compared to unlikely TB throughout the post-discharge period.
	Dewi et al. 2025	Indonesia	Comparative analytical design with a cross-sectional approach	Differences between the nutritional status of toddlers suffering from pulmonary tuberculosis before and after OAT treatment.
	Saraswati et al. 2025	Indonesia	Qualitative case-study design	Nutritional status declines due to decreased appetite, leading to weight loss; a balanced comparison was found between children with good and poor nutritional status.
	Amelia, 2024	Indonesia	Retrospective, cross-sectional study	Notable improvements in nutritional status after two months of treatment, with a decrease in malnutrition and an increase in good nutritional status; the type of TB and the presence of comorbidities influence the outcomes of nutritional status during anti-TB treatment.
	Umiijati, 2024	Indonesia	Cross-sectional study	TB in children was influenced by their nutritional status. Children's nutritional status can be improved by improving mothers’ ability to care for sick children.
	Singh et al. 2024	India	Multicentre cross-sectional study	189 (4.3%) had pulmonary TB.
	Sulistiyowati et al. 2024	Indonesia	Secondary data analysis from the 2018 Basic Health Research conducted by the Ministry of Health, Republic of Indonesia.	76.6% of children had at least one type of infectious disease. Children with poor nutritional status were contributed to the the likelihood of developing multiple types of infectious diseases.
	Singh et al. 2021	India	Multicentre study: retrospective chart review	10 of 56 children with SAM who have been screened were confirmed to have pulmonary TB.
	Tezol et al. 2019	Turkey	Retrospective cohort study	Nutrition status monitoring, correcting nutritional deficiencies and failures can be neglected in course of antituberculosis treatment
	Gurung et al. 2018	Nepal	Cross-sectional descriptive study	Food frequency, TB types, calorie intake, and nutritional status at the time of registration were significantly associated with the recent nutritional status of the participants.
	Chiang et al. 2016	Peru	Retrospective cohort study	Independent predictors of death/treatment failure were severe disease and WAZ ≤ −1.

	First author, year	Setting/ Country	Design	Outcomes
Food intake	Ghosh et al. 2026	India	Longitudinal field-based study	The food diary indicated that children (n=21) missed an average of one meal per day, particularly protein and green vegetables.
	Tsabedze, 2024	Eswatini	Qualitative study following a narrative design	Children feeding practices by most of the caregivers was categorized as unbalanced diet, facing the problem for inadequate supply of the ready-to-use therapeutic food and sharing of prescribed food supplies with other healthy children.
	Sulistiyowati et al. 2024	Indonesia	Secondary data analysis from the 2018 Basic Health Research conducted by the Ministry of Health, Republic of Indonesia.	Handwashing practices, immunization status, vitamin A supplementation, supplemental food, and child nutrition status were associated with the development of multiple types of infectious diseases.
	Oktofani, 2021	Indonesia	Case report	There are changes in behavior and knowledge of healthy life and dietary habit in patients and families of patients after an intervention based on evidence-based medicine that is patient-centered, family approach:<br>• Patient's diet does not implement a balanced nutritional diet,<br>• Patient does not like vegetables and fruits, only likes certain types of fruit.<br>• Patient and family was given counseling regarding balanced nutritional diets and high-calorie, high-protein diets for patients
	Franke et al. 2014	Peru	Case-control study	Consumption of fruits or vegetables ≥5 days per week was associated with nearly a 60% lower disease rate after adjusting for meat and fish intake.
Micronutrient supplementation	Khairiyadi et al. 2025	Indonesia	Prospective cohort design, a double-masked, individually randomized, placebo-controlled trial.	The treatment group (1.000 IU daily supplementation of cholecalciferol) exhibited a mean increase in body weight of 2.48 kg, height of 4.39 cm, a hemoglobin level of 0.15 mg/dl, and vitamin D level of 5.97ng/ml.
	Sulistiyowati et al. 2024	Indonesia	Secondary data analysis from the 2018 Basic Health Research conducted by the Ministry of Health, Republic of Indonesia.	Handwashing practices, immunization status, vitamin A supplementation, supplemental food, and child nutrition status were associated with the development of multiple types of infectious diseases.
	Middelkoop et al. 2023	South Africa	Multicenter phase 3 double blind randomized placebo-controlled trial	Weekly oral supplementation with 10,000 IU vitamin D 3 for 3 years elevated serum 25(OH)D concentrations among QFT-Plus-negative Cape Town schoolchildren but did not reduce their risk of QFT- Plus conversion.

	First author, year	Setting/ Country	Design	Outcomes
	Tamara et al. 2022	Indonesia	Randomized double-blind controlled trial	Vitamin D is beneficial in improving fever and cough resolution, and improving nutritional status in children with pulmonary TB and vitamin D insufficiency
	Ganmaa et al. 2020	Mongolia	Randomized controlled trial	Vitamin D supplementation did not result in a lower risk of TB infection, TB disease, or acute respiratory infection than placebo among vitamin D–deficient schoolchildren in Mongolia
	Olofin et al. 2014	Peru	Randomized controlled trial	Multivitamin supplementation did not significantly alter the risk of active TB.
	Lodha et al. 2014	India	Randomized, double-blind, placebo-controlled trial	Micronutrient supplementation did not modify the weight gain or clearance of lesions on CXR, but may improve height gain in children with intrathoracic TB in children.
	Nenni et al. 2013	Indonesia	Quasi- experimental study	Both groups had a significant increase in BMI (*p*=<0.001) post-treatment compared to pre-treatment. Supplementation with zinc, vitamin A and fish oil is associated with a significant increase in leptin levels and a significant decrease in TNF-a levels among children treated for TB
**Nutrition, in general**	Salazar-Austin et al. 2024	Ethiopia	Formative qualitative research, a cross-sectional design	TB services be integrated into other home-based services, including HIV, nutrition, and vaccination services to reduce workload on the already overstretched health extension workers.
**Guidelines**	WHO, 2013	Geneva	Practice guideline	World Health Organization. Guideline: Nutritional care and support for patients with tuberculosis
	WHO, 2025	Geneva	Practice guideline	WHO consolidated guidelines on tuberculosis. Module 1. Tuberculosis surveillance
